# Data on microRNA transcriptomic signatures, predicted gene targets and pathway analysis in response to cetuximab or panitumumab in colorectal cancer cells

**DOI:** 10.1016/j.dib.2026.112753

**Published:** 2026-04-16

**Authors:** Laetitia Corset, Frédéric Picou, Frauke Beilstein, Sajida Ibrahim, Romain Chautard, Sylviane Marouillat, Olivier Hérault, Thierry Lecomte, William Raoul

**Affiliations:** a UMR CNRS INRAE 7247 PRC « Physiology of Reproduction and Behavior », France; b Université de Tours, Inserm UMR 1069 N2COx « Niche, Nutrition, Cancer and Oxidative metabolism », France; c CHRU de Tours, Biological Hematology Department, France; d McGill University Health Centre, Montréal, QC, Canada; e CHRU de Tours, Department of Hepatology, Gastroenterology, and Digestive Oncology, France; f Université de Tours, Inserm UMR 1253 iBraiN, « Imaging Brain & Neuropsychiatry », France; g Plateforme ASB, Université de Tours, CHRU Tours, Inserm US 61, Tours, France

**Keywords:** Monoclonal antibodies, EGFR, Next-generation sequencing, miRNA, Resistance, Digestive cancer

## Abstract

Resistance to anti-epidermal growth factor receptor (EGFR) monoclonal antibodies (mAb) is common in metastatic colorectal cancer (mCRC) and reliable predictive biomarkers remain lacking. The identification of new predictive biomarkers is therefore necessary to assess the efficacy of anti-EGFR mAb, in order to optimize the therapeutic strategy for mCRC. Micro-RNAs (miR) are small non-coding RNAs that primarily regulate gene expression, and whose dysregulation in anti-EGFR resistance has been widely reported in CRC. Following a previous analysis using a miRnome strategy, the present study focused on NGS (next-generation sequencing) to investigate a broader range of miRNAs. We identified several miR resistance signatures as well as potential target genes and associated pathways following exposure to different doses of cetuximab or panitumumab in colorectal cancer cells. In the context of the search for useful biomarkers for this cancer, this could help optimize the design of *in vivo* experiments or future prospective clinical trials.

Specifications Table

**Table:** 

Subject	Health Sciences, Medical Sciences & Pharmacology
Specific subject area	Global gene expression profiling of colorectal cancer cells treated with cetuximab or panitumumab in two representative colorectal cancer cell lines.
Type of data	Raw FASTQ files, processed data files (figures and tables) and R scripts
Data collection	Total RNA was extracted from HCT116 and HT29 cells incubated with anti-EGFR monoclonal antibody, cetuximab or panitumumab or under control conditions. miRNA libraries were constructed and sequenced as 75-bp single-end reads on an Illumina NextSeq 500/550 platform. Sequencing data were processed through quality control, adapter trimming, and alignment to the human reference genome, followed by transcript reconstruction, gene expression quantification, and differential expression analysis using established RNA-seq pipelines in R. Significantly expressed miRNAs common to the antibody treatments were analyzed for predicted and validated targets using miRDB and TargetScan, applying stringent score thresholds and retaining only targets identified in both databases.
Data source location	Tours University, Faculty of Medicine, Tours, France (Latitude 47.38°, Longitude 0.669°)
Data accessibility	Repository name: Raw data were submitted in Gene Expression Omnibus (GEO) Data identification number: Accession number GSE316424 Direct URL to data: To review GEO accession GSE316424: https://www.ncbi.nlm.nih.gov/geo/query/acc.cgi?acc=GSE316424
Related research article	Chautard et al. Panitumumab and cetuximab affect differently miRNA expression in colorectal cancer cells [1]

## 1 Value of the Data

• Differentially expressed miR induced by cetuximab or panitumumab, two monoclonal antibodies targeting EGFR, provide valuable preliminary data to support studies on miR as biomarkers in colorectal cancer progression.
• This dataset enables researchers to further assess targets and pathways mediated by cetuximab or panitumumab linked to resistance of colorectal cancer cells, stimulating the generation of novel hypotheses.
• Downregulation or Upregulation of newly identified miR associated with potential targets offers a pavement to explore and determine signatures of interest in metastatic colorectal cancer.
• Our data supplement existing work on the resistance of anti-EGFR treatment in colorectal cancer.

## 2 Background

Resistance to anti-epidermal growth factor receptor (EGFR) monoclonal antibodies (mAb) in metastatic colorectal cancer (mCRC) is frequent and biomarkers are lacking. The only validated factors for anti-EGFR resistance demonstrated in clinical practice are activating mutations in *K-RAS* and *N-RAS* genes [2]. However, since not all patients with anti-EGFR have a positive survival outcome, it is essential to identify new biomarkers predictive of anti-EGFR efficacy to optimize the treatment strategy for mCRC.

Micro-RNAs (miR) are small non-coding RNAs that primarily regulate gene expression. Deregulation of miR expression in anti-EGFR resistance has been largely reported in CRC [3,4]. Given the heterogeneity of results and the complexity of elements influencing miR expression it remains difficult to understand the miR impact on the efficacy of anti-EGFR mAb [[5], [6], [7], [8]].

In a previous study using a miRnome strategy [1], we highlighted miR candidates and proved the feasibility of detection by measuring their expression in serum samples from mCRC patients. To analyze a broader spectrum of miR, we performed NGS (next-generation sequencing) screening to characterize the effects of cetuximab or panitumumab exposure in representative colorectal cancer cell lines. This screening strategy could serve as a basis for identifying miRs as potential biomarkers or signatures of resistance.

## 3 Data Description

HCT116 or HT29 CRC cells were treated with either 10 or 100 μg/ml cetuximab or panitumumab or without antibody for 48 h, then total RNA (small and large RNA) was isolated from samples for sequencing.

**Fig. 1:**
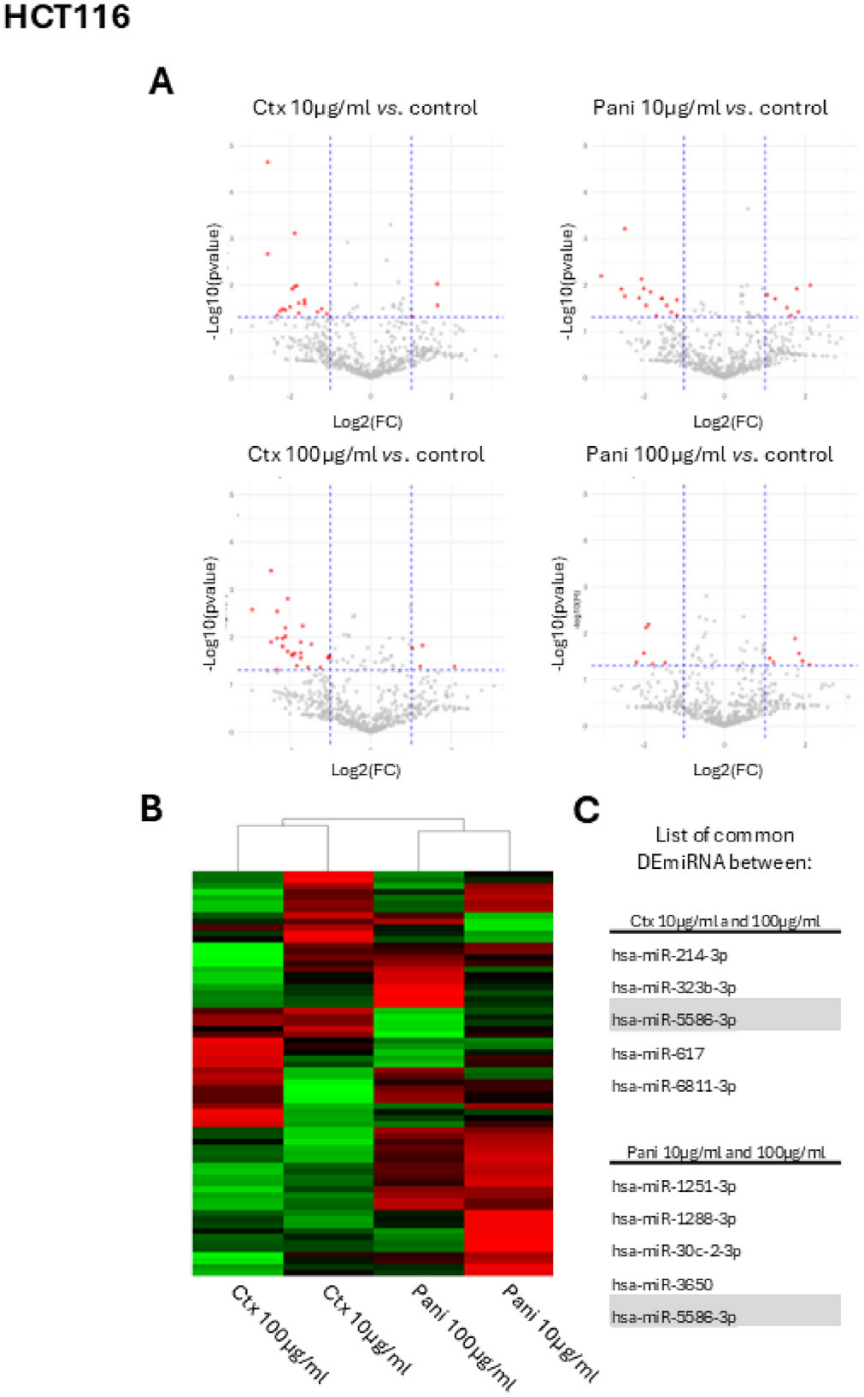
Study of HCT116 cells after anti-EGFR treatment *in vitro*. A. Volcano plot representation of miR expression in HCT116 cells following cetuximab (left panel) or panitumumab (right panel) exposure (10 or 100 µg/ml) compared to control condition, showing differentially expressed miRNA (DE miRNA) with |log2(fold change)| ≥1 and adjusted q-value < 0.05. Red labels represent significantly downregulated or upregulated miRNA. B. Heatmap highlighting effects of drug on HCT116 cells using unsupervised hierarchical representation. C. List of common DE miRNA between cetuximab treated cells (upper panel) or panitumumab treated cells (lower panel). Note that has-miR-5586–3p is common between all experimental conditions. Ctx: cetuximab; Pani: panitumumab; FC: Fold Change.

In a first step, regarding HCT116 cells only (Fig. 1A, left panel), treated with 10 µg/ml of cetuximab, 21 miR are differentially (ǀlog2(FC)ǀ>1) and significantly (q-values<0.05) altered and only three are overexpressed (Fig. 1B) in comparison to control. In case of 100 µg/ml of cetuximab treatment in the same cells, 28 miR are of interest regarding the previous threshold, but only 4 miR are upregulated, notably miR-3183 and miR-4707 (Fig. 1A, left panel). Note that few miR are significantly altered with both doses of treatments, including miR-214–3p, miR-323–3p, miR-5586–3p, miR-3650 and miR-5586–3p Regarding HCT116 at the 10 µg/ml concentration of panitumumab, 16 miR are over expressed and 7 down regulated (according with previously described criteria). For the 100 µg/ml concentration of panitumumab, 6 different miR are also over expressed (Fig. 1A, right panel) and 6 under expressed. Note that 5 miR are significantly altered with both doses of treatments, namely miR-1251–3p, miR-1288–3p, miR-30c-2–3p, miR-3650 and miR-5586–3p

Altogether, HCT116 cells results highlight some different miR altered in presence of different anti-EGFR mAb, without strong common signature between treatments (Fig. 1B), with only one miR (hsa-miR-5586–3p) that is dysregulated with both antibodies at two different concentrations. This miR could be further investigated as potential new biomarker of resistance to different anti-EGFR in *K-RAS* mutated CRC (Fig. 1C).

**Fig. 2:**
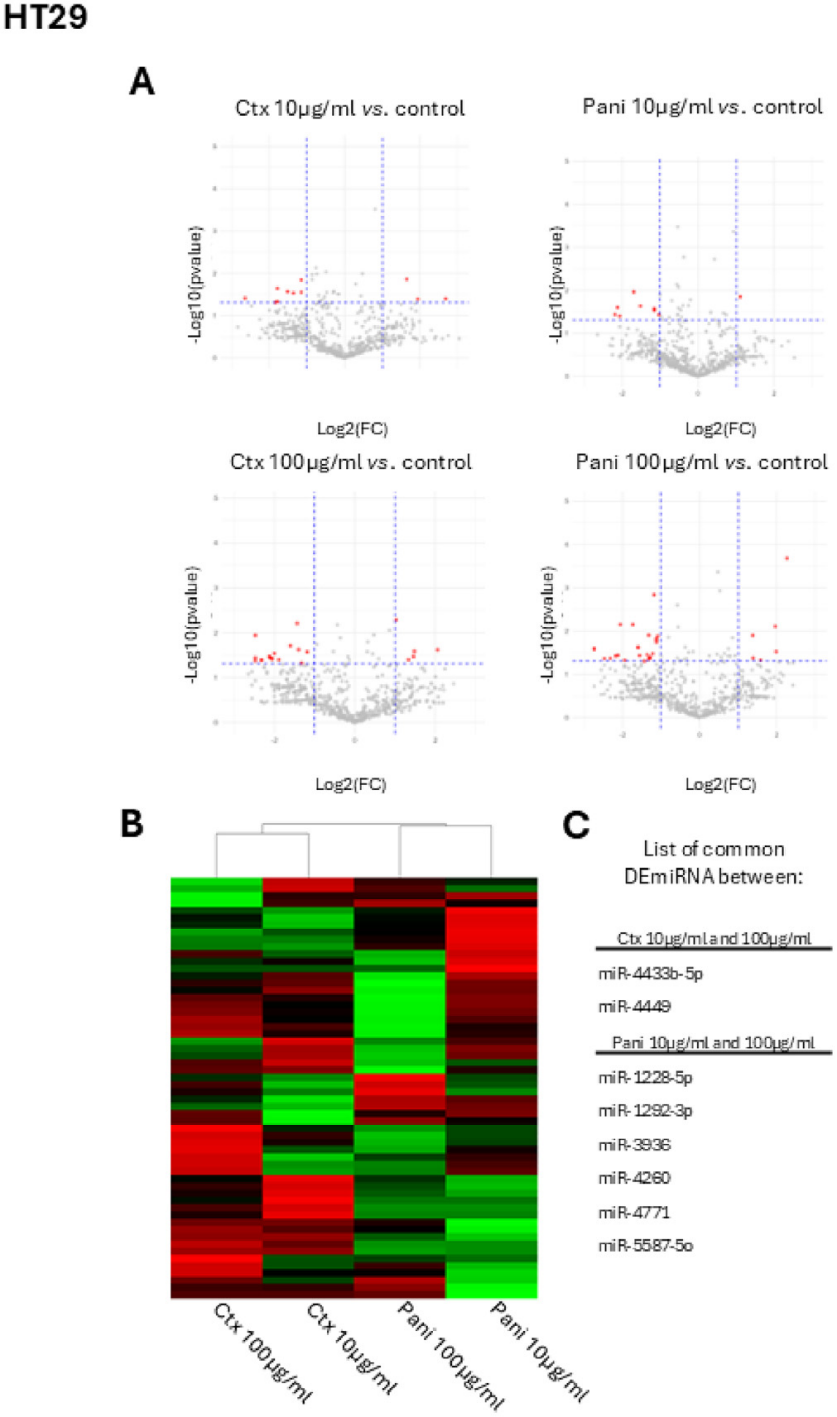
Study of HT29 cells after anti-EGFR treatment *in vitro*. A. Volcano plot representation of miR expression in HT29 cells following cetuximab (left panel) or panitumumab (right panel) exposure (10 or 100 µg/ml) compared to control condition, showing differentially expressed miRNA (DE miRNA) with |log2(fold change)| ≥1 and adjusted q-value < 0.05. Red labels represent significantly downregulated or upregulated miRNA. B. Heatmap highlighting effects of drug on HT29 cells using unsupervised hierarchical representation. C. List of common DE miRNA between cetuximab treated cells (upper panel) or panitumumab treated cells (lower panel). Ctx: cetuximab; Pani: panitumumab; FC: Fold Change.

In a second step, regarding HT29 cells (Fig. 2), which are more likely to be altered by anti-EGFR treatment, cells treated with 10 µg/ml of cetuximab show 3 up-regulated and 8 down-regulated miR in comparison to control. In case of 100 µg/ml of cetuximab treatment in the same HT-29 cells, 5 are overexpressed and 15 underexpressed. Crossing these lists, only two miR are misregulated in both doses of cetuximab treatment, miR-4433b-5p and miR-4449 in our model. Regarding panitumumab treatment (10 µg/ml), only one miR is overexpressed and 9 miR under regulated. At 100 µg/ml of panitumumab, 6 miR are overexpressed and 24 are under expressed, indicating a dose effect response for this drug in this model (Fig. 2A and B). Note that 6 common DE miRNA were identified between 10 µg/ml and 100 µg/ml of panitumumab (Fig. 1C), but no common signature with cetuximab was found.

**Fig. 3:**
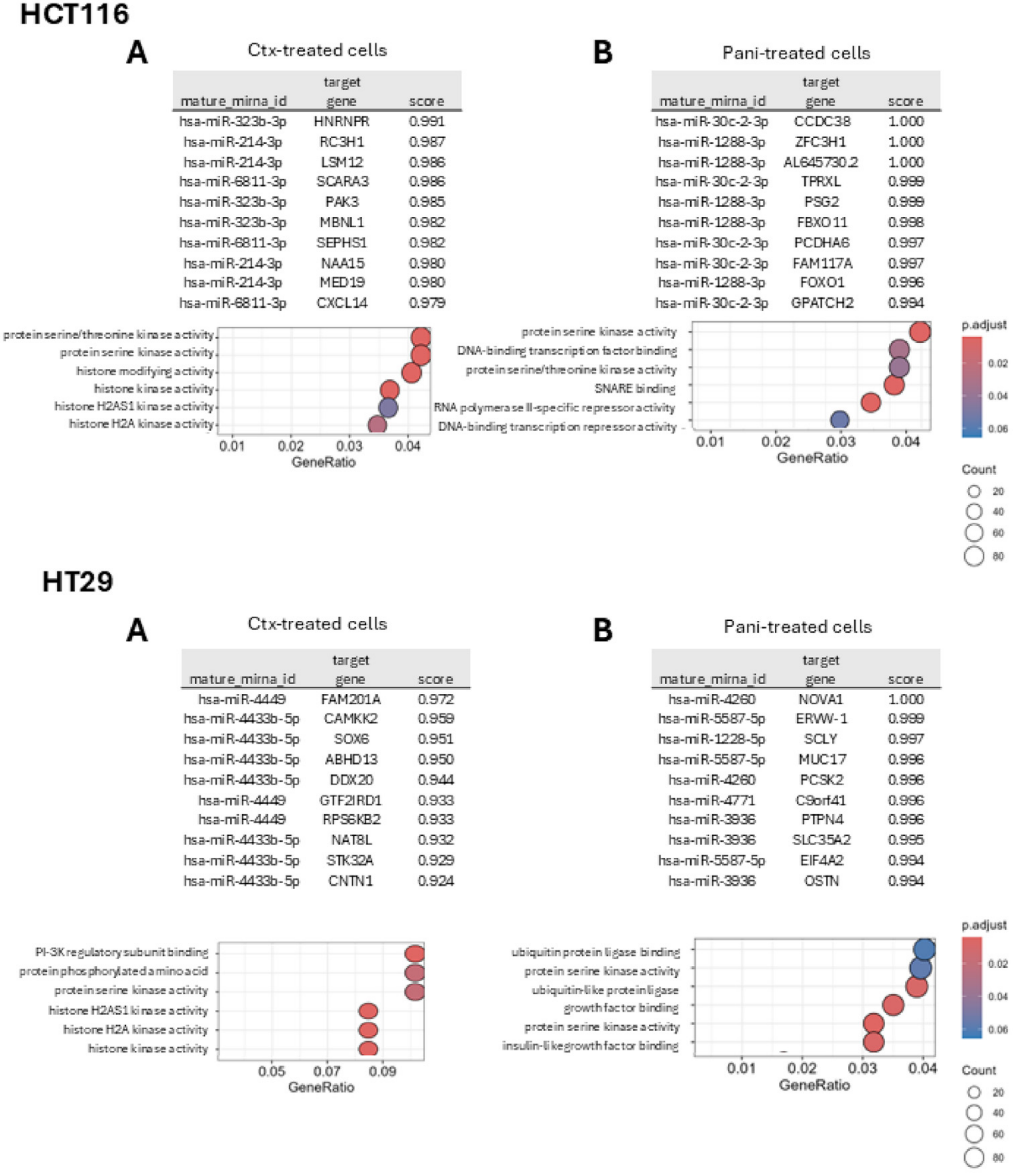
Pathway implicated in miR-targeted by cetuximab or panitumumab. *Upper panels:* Top ten potential target genes linked to the identified common miR after NGS screening in HCT116 cell line following cetuximab (A) or panitumumab (B) exposure and their associated pathways. *Lower panels:* Top ten potential target genes linked to the identified common miR after NGS screening in HT29 cell line following cetuximab (A) or panitumumab (B) exposure and their associated pathways. Ctx: cetuximab; Pani: panitumumab.

Furthermore, to elucidate potential target genes linked to dysregulated miR identified above after cetuximab or panitumumab treatment in both cell lines, Enrichr analysis was performed (Fig. 3, libraries are listed in Supplementary Table 3). Potential pathways for each group were illustrated under each treatment group using gene ratio representation, respectively for HCT116 and HT29 cell lines.

For HCT116 treated cells, protein serine kinase activity is the most redundant pathway targeted by miRNA in case of cetuximab or panitumumab treatment (10 or 100 µg/ml). For HT29, altered pathways are more complex, potentially reflecting the sensibility to anti-EGFR treatment. These pathways include protein serine kinase activity, PI3K activity, histone activity or growth factor function.

Overall, our data provided further evidence for the differential use of cetuximab and panitumumab in cancer diseases and especially in mCRC. Of note, cetuximab is a chimeric IgG1 antagonistic mAb that competitively targets the extracellular domain of EGFR. Cetuximab can also recruit effective cytotoxic immune cells, such as monocytes, macrophages, lymphocytes and natural killer cells, through its Fc portion. This binding with Fcγ receptors triggers the cytolytic activity mediated by perforins and granzymes, or by the Fas ligand (FasL) or tumor necrosis factor–related apoptosis-inducing ligand (TRAIL). This immune mechanism is called antibody-dependent cellular cytotoxicity (ADCC). Panitumumab is a human IgG2 antagonistic mAb with a mechanism of action similar to that of cetuximab, except for its limited ADCC. Interestingly Gomes *et al*. [9] showed that miR-143 or miR-145 overexpression increased cell sensitivity to cetuximab, resulting in a significant increase of ADCC, independently of KRAS status. However, to date, very little data is available regarding ADCC and miR in CRC.

The main characteristics of the two anti-EGFR mAbs have been clearly illustrated and discussed in the recent review by Garcia-Foncillas *et al*. [10]. The authors concluded that these two distinct therapeutic molecules must be carefully considered individually when planning treatment, particularly in the case of mCRC. To illustrate this conclusion, the innovative EXONERATE study (EXOsome and cell-free miRNAs of anti-EGFR ResistAnce) identified a miR profile obtained via exosome-based liquid biopsy that can predict depth of response and survival outcome following administration of cetuximab or panitumumab in mCRC [11].

Finally, in order to circumvent acquired resistance, dual targeting of the EGFR pathway, new molecular formats or combination therapies could serve as alternative strategies [[12], [13], [14], [15]]. These new treatment regimens, once clinically validated, will also require appropriate biomarkers.

## 4 Conclusions

In our study, we identified miR signatures, which, to the best of our knowledge, had not yet been described in the literature and that are implicated in treatment resistance following dose-gradient treatments with cetuximab or panitumumab in CRC. In addition, we identified potential target genes and associated pathways, highlighting kinase activities as a central regulatory hub. In a context of useful biomarkers for this cancer, this could help optimize the design of *in vivo* experiments, for example using xenografted models, or in future prospective clinical trials.

## 5 Experimental Design, Materials and Methods

### 5.1 Cell culture

HCT116 and HT29 hCRC cell lines were purchased from the American Type Culture Collection. HT29 is a *K-RAS* unmutated / *B-RAF* mutated moderately-sensitive cell line to cetuximab and HCT116 is a K-RAS mutated highly-resistant cell line [16,17].

Cells were cultured in DMEM medium (Thermo Fisher, Waltham, MA, USA) supplemented with 10 % fetal bovine serum (FBS, Thermo Fisher) They were maintained at 37 °C in a humidified atmosphere of 5 % CO_2_. Cells were incubated 48 h with either cetuximab or panitumumab (10 µg/ml or 100 µg/ml), or with buffer without antibody (control condition), one day after plating at a seeding of 600 000 cells / well in 6-well plates. These experiments were replicated 4 times for each condition.

### 5.2 RNA-sequencing and data analysis

Total RNA was extracted using the NucleoZOL RNA purification kit (Macherey-Nagel, Dûren, Germany) and quantified with a NanoDrop spectrophotometer (Thermo Fisher). RNA quality and integrity were assessed using the RNA 6000 Nano Kit on an Agilent 2100 Bioanalyzer (Agilent Technologies, Santa Clara, *CA*, USA). miRNA libraries were prepared from 100 ng of total RNA with QIAseq miRNA Library Kit / QIAseq miRNA 48 Index (Qiagen, Venlo, Netherland). Libraries were pooled and prepared according to the denaturing and diluting libraries according to manufacturers (Illumina,San Diego, *CA*, USA) . Single‐end sequencing was carried out on NextSeq 500/550 High Output Kit v2.5 (75 Cycles) kit (Illumina).

After base call conversion and demultiplexing with bcl2fastq (v2.20.0.422; Illumina), read quality was assessed using FastQC (v0.11.9). Illumina adapter were trimmed and reads with Phred quality score below 25 were filtered. Filtered reads were aligned to the human reference genome (Ensembl release 107) using HISAT2 (v2.2.1). Transcript reconstruction was performed with StringTie, and novel transcripts were identified using Cuffcompare, a component of the Cufflinks package. Novel protein-coding transcripts were merged with reference annotations, and reads were remapped using Bowtie2.

Gene expression levels were quantified using RNA-Seq by Expectation Maximization (RSEM) and reported as transcripts per million (TPM). Differential gene expression analysis was assessed using DESeq2, with results reported as log₂ fold-change values. Genes with an average expression ≥10 TPM were retained, and differential expression was defined as |log₂ fold change| > 1 with an adjusted *q*-value < 0.05. miRNA differential expression analysis was performed using DESeq2 according to Love et *al*. [18]. The two lists of DE miRNA are presented in supplementary material: Supplementary Table 1_DEmiRNA_HCT116all and Supplementary Table 2_DEmiRNA_HT29all. Statistical analyses and data visualization were performed using R version 3.6.1. All codes used for analyses are available in: https://github.com/picoufrederic/Colon-cancer-WR.git

For each antibody stimulation and cell line, miRNAs significantly expressed at both tested concentrations compared with control cells were selected. Predicted and validated miRNA targets were identified using freely available databases, miRDB and TargetScan v7.2, selected for their robustness and widespread use [19,20]. Only miRNAs common to both antibody concentrations were retained. Stringent filtering criteria were applied, including a target score ≥90 % in miRDB and a cumulative weighted context score ≥60 % in TargetScan. Target genes were retained only if identified in both databases. Codes are available in supplementary data. Enrichr analysis was performed to characterize identify potential impacted pathways linked to miR profiling after cetuximab or panitumumab treatment in both cell lines [[21], [22], [23]] (Supplementary Table 3).

## Limitations

*None*.

## Ethics Statement

The authors have read and follow the ethical requirements for publication in Data in Brief and confirm that the current work does not involve human subjects, animal experiments, or any data collected from social media platforms.

## Credit Author Statement

**LC**: conceptualization, investigation, data curation, visualization, formal analysis, validation, writing –original draft, writing –review & editing, methodology ; **FP**: data curation, visualization, formal analysis, validation, writing –original draft, writing –review & editing, methodology, data upload ; **FB**: data curation, formal analysis, validation, writing –review & editing, data upload ; **SI**: visualization, formal analysis, writing –review & editing ; **RC**: formal analysis, writing –review & editing ; **SM**: visualization, formal analysis, data curation, writing –review & editing ; **OH**: investigation, validation, writing –review & editing, supervision, funding acquisition ;**TL**: conceptualization, writing –original draft, writing –review & editing, supervision, funding acquisition ; **WR**: conceptualization, data curation, visualization, formal analysis, validation, writing –original draft, writing –review & editing, methodology, supervision, funding acquisition.

## Data Availability

Gene Expression Omnibus GEO - Series GSE316424Cetuximab or panituximab differentially impact miRNA expression and potential signaling pathway in cancer colon HCT116 or HT29 cells in vitro (Original data).

## Appendix Supplementary materials

Image, application 1

Image, application 2

Image, application 3

## References

[1] Chautard R., Corset L., Ibrahim S., Desvignes C., Paintaud G., Baroukh N. (2021). Panitumumab and cetuximab affect differently miRNA expression in colorectal cancer cells. Biomark. Med..

[2] Lievre A., Bachet J.B., Boige V., Cayre A., Le Corre D., Buc E. (2008). KRAS mutations as an independent prognostic factor in patients with advanced colorectal cancer treated with cetuximab. J. Clin. Oncol..

[3] Garajova I., Ferracin M., Porcellini E., Palloni A., Abbati F., Biasco G. (2017). Non-coding RNAs as predictive biomarkers to current treatment in metastatic colorectal cancer. Int. J. Mol. Sci..

[4] Madurantakam Royam M., Kumarasamy C., Baxi S., Gupta A., Ramesh N., Kodiveri Muthukaliannan G. (2019). Current evidence on miRNAs as potential theranostic markers for detecting chemoresistance in colorectal cancer: a systematic review and meta-analysis of preclinical and clinical studies. Mol. Diagn. Ther..

[5] Cappuzzo F., Sacconi A., Landi L., Ludovini V., Biagioni F., D'Incecco A. (2014). MicroRNA signature in metastatic colorectal cancer patients treated with anti-EGFR monoclonal antibodies. Clin. Colorectal. Cancer.

[6] Mlcochova J., Faltejskova P., Nemecek R., Svoboda M., Slaby O. (2013). MicroRNAs targeting EGFR signalling pathway in colorectal cancer. J. Cancer Res. Clin. Oncol..

[7] Ragusa M., Majorana A., Statello L., Maugeri M., Salito L., Barbagallo D. (2010). Specific alterations of microRNA transcriptome and global network structure in colorectal carcinoma after cetuximab treatment. Mol. Cancer Ther..

[8] Wei S., Hu W., Feng J., Geng Y. (2022). Promotion or remission: a role of noncoding RNAs in colorectal cancer resistance to anti-EGFR therapy. Cell Commun. Signal..

[9] Gomes S.E., Simoes A.E., Pereira D.M., Castro R.E., Rodrigues C.M., Borralho P.M. (2016). miR-143 or miR-145 overexpression increases cetuximab-mediated antibody-dependent cellular cytotoxicity in human colon cancer cells. Oncotarget..

[10] Garcia-Foncillas J., Sunakawa Y., Aderka D., Wainberg Z., Ronga P., Witzler P. (2019). Distinguishing features of Cetuximab and Panitumumab in colorectal cancer and other solid tumors. Front. Oncol..

[11] Xu C., Mannucci A., Esposito F., Oliveres H., Alonso-Orduna V., Yubero A. (2025). An exosome-based liquid biopsy predicts depth of response and survival outcomes to Cetuximab and Panitumumab in metastatic colorectal cancer: the EXONERATE study. Clin. Cancer Res..

[12] Rios-Hoyo A., Monzonis X., Vidal J., Linares J., Montagut C. (2024). Unveiling acquired resistance to anti-EGFR therapies in colorectal cancer: a long and winding road. Front. Pharmacol..

[13] Patnaik S.K., Swaroop A.K., Nagarjuna P., Nanjan M.J., Chandrasekar M.J.N. (2024). Peptides for dual targeting of ErbB1 and ErbB2: blocking EGFR cell signaling transduction pathways for cancer chemotherapy. Curr. Mol. Pharmacol..

[14] Joubert N., Beck A., Dumontet C., Denevault-Sabourin C. Antibody-drug conjugates: the last decade. Pharmaceuticals (Basel). 2020;13(9). 10.3390/ph13090245 PMC7558467 32937862

[15] Du Y., Karatekin F., Wang W.K., Hong W., Boopathy G.T.K. (2025). Cracking the EGFR code: cancer biology, resistance mechanisms, and future therapeutic frontiers. Pharmacol. Rev..

[16] Troiani T., Napolitano S., Vitagliano D., Morgillo F., Capasso A., Sforza V. (2014). Primary and acquired resistance of colorectal cancer cells to anti-EGFR antibodies converge on MEK/ERK pathway activation and can be overcome by combined MEK/EGFR inhibition. Clin. Cancer Res..

[17] Jhawer M., Goel S., Wilson A.J., Montagna C., Ling Y.H., Byun D.S. (2008). PIK3CA mutation/PTEN expression status predicts response of colon cancer cells to the epidermal growth factor receptor inhibitor cetuximab. Cancer Res..

[18] Love M.I., Huber W., Anders S. (2014). Moderated estimation of fold change and dispersion for RNA-seq data with DESeq2. Genome Biol..

[19] Chen Y., Wang X. (2020). miRDB: an online database for prediction of functional microRNA targets. Nucleic. Acids. Res..

[20] Agarwal V., Bell G.W., Nam J.W., Bartel D.P (2015). Predicting effective microRNA target sites in mammalian mRNAs. Elife.

[21] Xie Z., Bailey A., Kuleshov M.V., Clarke D.J.B., Evangelista J.E., Jenkins S.L. (2021). Gene set knowledge discovery with Enrichr. Curr. Protoc..

[22] Kuleshov M.V., Jones M.R., Rouillard A.D., Fernandez N.F., Duan Q., Wang Z. (2016). Enrichr: a comprehensive gene set enrichment analysis web server 2016 update. Nucleic. Acids. Res..

[23] Chen E.Y., Tan C.M., Kou Y., Duan Q., Wang Z., Meirelles G.V. (2013). Enrichr: interactive and collaborative HTML5 gene list enrichment analysis tool. BMC. Bioinformatics..

